# AI in the hands of imperfect users

**DOI:** 10.1038/s41746-022-00737-z

**Published:** 2022-12-28

**Authors:** Kristin M. Kostick-Quenet, Sara Gerke

**Affiliations:** 1 Center for Medical Ethics and Health Policy, Baylor College of Medicine, Houston, TX USA; 2 Penn State Dickinson Law, Carlisle, PA USA

**Keywords:** Ethics, Medical ethics

## Abstract

As the use of artificial intelligence and machine learning (AI/ML) continues to expand in healthcare, much attention has been given to mitigating bias in algorithms to ensure they are employed fairly and transparently. Less attention has fallen to addressing potential bias among AI/ML’s human users or factors that influence user reliance. We argue for a systematic approach to identifying the existence and impacts of user biases while using AI/ML tools and call for the development of embedded interface design features, drawing on insights from decision science and behavioral economics, to nudge users towards more critical and reflective decision making using AI/ML.

## Introduction

The use of artificial intelligence and machine learning (AI/ML) continues to expand in healthcare, with great promise for enhancing personalized clinical decision making^1^. As AI/ML tools become more widespread, much attention has been given to mitigating bias in algorithms to ensure they are employed fairly and transparently. However, less attention has fallen to mitigating potential bias among AI’s human users. As automated systems become more sophisticated in their capacity to predict, screen for, or diagnose disease, the temptation to rely on them in clinical decision making will increase^2^. However, factors that influence user reliance on AI are poorly understood, and healthcare professionals lack guidelines about the role that AI should play in their decision making. We argue for a more systematic approach to identifying the existence and impacts of user biases while using AI tools and their effects on clinical decision making and patient outcomes. Specifically, we call for greater empirical research into how to mitigate biases with anticipated negative outcomes through the use of embedded interface design features, drawing on insights from decision science and behavioral economics, to nudge users towards more critical and reflective decision making using AI tools.

### Expand notions of user testing

Recognizing the potential harms of overreliance on AI systems in the context of high stakes decision making, regulators and policymakers seem to endorse keeping humans “in the loop” and focus their action plans and recommendations on improving the safety of AI/ML systems such as through enhanced computational accuracy^3–5^. Meanwhile, developers are innovating new ways of addressing trustworthiness, accountability, and explainability of “black box” AI/ML that involves deep learning or neural nets with significant interpretability limitations^6,7^. These goals appear to be particularly important when using AI/ML in clinical decision making, not only because the costs of misclassifications and potential harm to patients are high but also because undue skepticism or lack of trust can reduce stakeholders’ adoption of promising new AI technologies and inhibit their use and availability outside of experimental settings.

One of us (SG in Babic et al.^8^), however, recently warned healthcare professionals to be wary of explanations that are presented to them for black box AI/ML models.

Explainable AI/ML … offers post hoc algorithmically generated rationales of black box predictions, which are not necessarily the actual reasons behind those predictions or related causally to them. Accordingly, the apparent advantage of explainability is a “fool’s gold” because post hoc rationalizations of a black box are unlikely to contribute to our understanding of its inner workings. Instead, we are likely left with the false impression that we understand it better.”

Consequently, instead of focusing on explainability as a strict condition for AI/ML in healthcare, regulators like the U.S. Food and Drug Administration (FDA) should focus more holistically on those aspects of AI/ML systems that directly bear on their safety and effectiveness—especially, how these systems perform in the hands of their intended users. While the FDA recently published its final guidance explicitly recognizing the risks of automation bias^9^ and is working on a new regulatory framework for modifications to AI/ML-based software as a medical device (i.e., software that is itself classified as a medical device under section 201(h)(1) of the U.S. Federal Food, Drug, and Cosmetic Act^10^), Babic et al. argue that regulators like the FDA should also, at least in some cases, emphasize well-designed clinical trials to test human factors and other outcomes of using AI in real-world settings. Gerke et al.^11,12^ similarly argue that more algorithmic tools must be prospectively tested to understand their performance across a variety of procedural contexts that mirror their intended use settings and human-AI interactions. The type of user testing these scholars are suggesting goes beyond the typical usability and acceptability testing that characterizes the pipeline from *beta* to a more finalized version of an AI tool. That type of testing is most often done heuristically^13^, using a small set of evaluators to examine the interface and judge its compliance with relevant usability principles (e.g., interpretability, perceived utility, navigability, satisfaction with use, etc.). While these metrics are often useful for gauging proximate user experiences (i.e., “UX” testing) with a tool’s interface, a deeper level of user testing is needed^14^ to help identify and address potential sources of “emergent” or “contextual” bias^15^ that arise due to mismatches between a product’s design and the characteristics of its users, use cases or use settings. These mismatches may be more difficult to predict and account for in the case of AI tools than for traditional medical devices or pharmaceuticals whose performance is less contingent on user interactions and interpretations^12^, or whose adaptive algorithms continuously change^16^. Mitigating these mismatches can only be achieved by broadening our notion of user testing beyond its current focus on AI performance metrics and proximate usability to examine human and systemic factors shaping how AI systems are applied in practice^17,18^ by imperfect users in imperfect settings. Further, testing does not have to be limited to simply observing how individuals in various contexts interact with AI tools; we can also test how best to *shape* those interactions using existing insights from the behavioral sciences, as we discuss below.

### Trust in the eye of the (imperfect) beholder

At this stage in the history of human-machine relations, nearly everyone is an imperfect user of AI. By this, we mean imperfectly rational: our interpretations and integration of information into decision making, including insights derived from AI, are susceptible to well-documented forms of bias^19,20^. Not all biases, however, are equally salient or relevant to the safe, effective, and responsible use of AI. From both legal and ethical perspectives, the most important cognitive biases are those that impact the extent to which humans rely on AI in their decision making in ways that introduce risk. Reliance falls along a spectrum of utter rejection or skepticism of AI on one end to “blind” overreliance or acceptance of AI-derived conclusions on the other. Both types of error can have negative impacts on patient outcomes, with underreliance potentially leading to errors of omission and overreliance on errors of commission.

Where clinical decision makers fall along this spectrum depends on how much they trust an AI system. Literature from anthropology and developmental psychology documents findings that human trust is influenced by how other people behave in contexts of reciprocity and exchange^21^, not only of goods and services but also attachment behaviors^22,23^ (e.g., affection, nurturance). Loyalty^24^, integrity^25^, and competence^26^ play important roles in human-human trust, increasingly conceptualized as an evolved capacity to help us navigate complex social dynamics and to mitigate personal risk by understanding which entities and objects can be trusted under which contingencies^27–29^. While we know a great deal about trust in human relationships, we are just beginning to understand how and in what circumstances humans trust machines. Literature on human-machine interactions, or “human factors” research, has existed for decades in other domains, including military, aerospace, and robotics; but only within the last decade have questions surrounding human interactions with autonomous systems (e.g., automation bias) begun to animate the field of AI broadly, and AI ethics in particular^2,11^.

### Impacts of uncertainty and urgency on decision quality

Trust plays a particularly critical role when decisions are made in contexts of uncertainty. Uncertainty, of course, is a central feature of most clinical decision making, particularly for conditions (e.g., COVID-19^30^) or treatments (e.g., deep brain stimulation^31^ or gene therapies^32^) that lack a long history of observed outcomes. As Wang and Busemeyer (2021)^33^ describe, “uncertain” choice situations can be distinguished from “risky” ones in that risky decisions have a range of outcomes with known odds or probabilities. If you flip a coin, we know we have a 50% chance to land on heads. However, to bet on heads comes with a high level of risk, specifically, a 50% chance of losing. Uncertain decision-making scenarios, on the other hand, have no well-known or agreed-upon outcome probabilities. This also makes uncertain decision making contexts risky, but those risks are not sufficiently known to the extent that permits rational decision making. In information-scarce contexts, critical decisions are by necessity made using imperfect reasoning or the use of “gap-filling heuristics” that can lead to several predictable cognitive biases^20^. Individuals might defer to an authority figure (messenger bias^34^, authority bias^35^); they may look to see what others are doing (“bandwagon” and social norm effects^35,36^); or may make affective forecasting errors, projecting current emotional states onto one’s future self^37^. The perceived or actual urgency of clinical decisions can add further biases, like ambiguity aversion (preference for known versus unknown risks^38^) or deferral to the status quo or default^39^, and loss aversion (weighing losses more heavily than gains of the same magnitude^40^). These biases are intended to mitigate risks of the unknown when fast decisions must be made, but they do not always get us closer to arriving at the “best” course of action if all possible information were available.

### Reducing or exacerbating uncertainty

One of AI’s most compelling advantages for healthcare is to reduce this uncertainty—for example, by calculating a personalized estimate that a patient’s condition will worsen after X amount of time or will enjoy a survival benefit of Y number of years post-intervention. However, whether AI successfully contributes to reducing uncertainty still depends to a large extent on how estimates are interpreted and acted upon. A small number of studies examining decisional biases when using AI have identified that physicians across expertise levels often fail to dismiss inaccurate advice generated by computerized systems (automation bias^41–45^), but as well as by humans, indicating that people are generally susceptible to suggestions. The tendency to follow even bad advice appears to be even more prevalent among participants with less domain expertise^46,47^. Receiving such advice from AI systems can raise further dangers by potentially engaging other cognitive biases such as anchoring effects and confirmatory bias, in which users are primed towards a certain perspective and disproportionately orient their attention to information that confirms it^48^. Other studies have found that participants are averse to following algorithmic advice when making final decisions (algorithmic bias)^49–51^, but this result is inconsistent with other studies, which show people sometimes prefer algorithmic to human judgment^46,47,52^.

Given the diversity of cognitive biases and contingencies under which they are likely to emerge, further systematic research is needed to document which salient factors shape how we integrate AI into decisions and how best to calibrate trust so that it matches what AI systems can actually do (e.g., predict something with a given degree of probability and accuracy). In robotics, poor “trust calibration” between humans and machines is viewed as a core vulnerability and key predictor of performance breakdown^53,54^. Likewise, putting AI in the hands of users without systematically measuring, controlling for, or otherwise trying to calibrate trust and reliance likely exacerbates rather than reduces the already high levels of uncertainty that characterize these decision-making contexts, with potentially grievous consequences.

### The uncertain role of AI in clinical decision making

The current push^55–57^ to enhance healthcare professionals’ literacy in AI/ML highlights a need to replace idiosyncratic variation with informed reasoning about the role that AI should play in clinical decision making. However, it is hard to know what kind of guidance healthcare professionals should receive when so few empirical conclusions have been drawn about how AI is or should be used in clinical (or any) decision making. Taking lessons from algorithmic tools that have been shown to reproduce negative societal biases in predicting factors like criminal recidivism^58^, health status and insurability^1^, and disease (e.g., skin cancer) risk^59^, many scholars argue^60,61^ that AI tools should not replace any decisions that are considered “high stakes”—those with significant health or justice-related impacts. In the healthcare setting, some experts recommend that even AI with a well-demonstrated capacity to autonomously identify and diagnose disease should be confirmed with human-led testing^62,63^. Similar conclusions have been made about autonomous weapons systems (AWS) in military^64^ and maritime (e.g., unmanned shipping^65^) applications, with ongoing debates about whether to keep humans “in” the loop or “on” the loop, the latter suggesting that humans may not need to take an active role in decision making but can (and should) still intervene or be able to appeal to AI inferences when their conclusions contradict those of the AWS (if caught in time).

If we agree that humans should still be “in” or “on” the loop, how should one expect healthcare professionals to react to AI-derived information? The recommendation to proceed with caution, while warranted, seems too broad to fit the decisional needs of physicians engaging powerful AI to inform complex medical decisions. There is growing agreement that proficiency in AI (including its shortcomings related to bias, transparency, and liability) should be part of medical education, with suggestions that medical students must acquire sufficient knowledge of data science, biostatistics, computational science, and even health AI ethics^66^ to ensure they can, among other things, separate “information from hype” and critically evaluate AI systems^57,67^. Others^68^ have argued that learning effective debiasing strategies and cultivating awareness of how heuristics can impact clinical decision making should be prioritized in all stages of medical education. However, it remains unclear which biases healthcare providers should be made most aware of; whether providers should be responsible for being aware of their own biases, or whether bias mitigation may (or should) be embedded in standardized processes for implementing AI tools in clinical decision making or in the design of the technologies themselves.

### Enhancing decision quality by design

While it is likely true that physicians will increasingly need to learn how to responsibly use AI to keep pace with clinical innovations, other complementary approaches should also be explored. One promising option is to support physicians in their likelihood to demonstrate the specific characteristics we value in clinical decision making by embedding bias mitigation techniques into the very design features of our AI systems and user interfaces. This notion builds on longstanding work in computing ethics^69,70^ and is known by various terms, including Value-Sensitive Design (VSD^71^), Values @ Play^72^, reflective design^73^, adversarial design^74^, and critical technical practice^75^, and was originally pioneered by Friedman and Nissenbaum^76,77^ in the 1990s as a way to encourage a reflective, iterative process for shaping human-computer interactions that prioritize trust and user welfare. A great deal of variation remains in how VSD is carried out, but the centrally motivating assumption behind this approach is that reflective design approaches can help to mitigate user biases for more favorable outcomes. Following the three main stages of VSD would entail identifying the range and diversity of stakeholder values and how best to balance them towards an articulated goal (*conceptual*), observing impacts of given values and practices on relevant outcomes (*empirical*), and devising technical specifications to design systems that reflect or help to shape the use of a system to align with stakeholders’ values (*technical*). An example would be to design interactive web browser cookie management systems to reflect principles of privacy, voluntariness, and right to disclosure^71^. Scholars have extended a fourth and ongoing step of *life-cycle monitoring* and evaluation to VSD for AI specifically, given the often unforeseeable impacts and adaptive nature of AI tools^14,78^.

Building on these approaches, we argue that a VSD approach could not only help to embed values into the design of AI tools but also to actively and strategically influence (nudge) users to engage in more ethical and critical reflection in their use of such tools. Such an approach requires critical engagement with the ethics of nudging in health decisions as well as identification of the range of target values one wants physicians to demonstrate in decision making. Nudging is a form of libertarian paternalism in which decisions are actively shaped through strategies such as information framing, structuring incentives, and other means to enhance the uptake of certain behaviors^79^. While evidence for the efficacy of this approach dates back nearly two decades^80^, nudging tactics have shown to be effective, for example, during the COVID-19 pandemic to encourage compliance with public health-promoting behaviors, such as handwashing and social distancing^81^. Though not without its critiques (e.g., that it can be a form of manipulation^82,83^), a central rationale of nudging is to preserve individual choice while guiding people toward behaviors with population-level benefits^84^. However, determining who gets to decide which values are engaged in service of making “good” decisions when using an AI tool is complex and should draw on perspectives from multiple, diverse stakeholders, not just those of developers designing these systems. The Hippocratic Oath establishes a fundamental criterion that physicians’ decisions should be in service of what they believe to be a patients’ best interests. Additional criteria come from a rich literature on decision making and clinical decision support^85^, suggesting that “quality” decisions are those that are informed and generate positive outcomes that are congruent with a patient’s values. Other target values, such as decisional autonomy^82^, are likely to be relevant, and it should be noted that salient target values may shift depending on the nature of the AI tool or the ethical issues raised by its intended users or use contexts. For example, an AI tool designed to predict and prevent onset of psychiatric illness in adolescents raises a particular set of target values in decision making (e.g., decisional autonomy, patients’ right to an open future) while a tool to identify presence and prognosis of lung cancer in adults may raise others (e.g., avoidance of negative emotional reactions, actionability considerations, patients’ right to not know). Research is needed to elucidate which target values for “quality” decision making are most salient in which clinical scenarios.

### AI interfaces that encourage critical reflection

One target value that is likely to be relevant in all clinical decision making involving AI is the need to promote reflexivity in decision making in order to avoid the potential negative consequences of overreliance on AI. A growing literature^1,86^ demonstrating the potentially deleterious effects of overreliance on AI algorithms highlights the importance of reflexivity and deliberation as guiding principles for AI deployment. These explorations and observations thus inform the conceptual and empirical stages of the VSD approach, leaving the technical challenge of designing interfaces that will help to shape the deliberative and reflexive use of AI systems in ways that align with users’ interests. Research has demonstrated that the ways in which information is presented can influence users’ likelihood of engaging in reflective or critical thought. For example, a study by Zhang et al.^87^ employed a simple interface nudge to encourage reflection by asking participants to answer brief questions clarifying their own opinions versus what they considered to be reasons driving alternative perspectives. Weinmann^88^ developed an online interface with similar questions to enhance “deliberation within” by asking questions that encouraged reasoning about alternative perspectives. Other research by Harbach et al.^89^ demonstrates the effectiveness of using interface design elements to inspire reflection by illustrating consequences of user choices (e.g., reminding users of the potential impacts on selecting certain user privacy parameters). Menon et al.^90^ similarly explored how modifying “interface nudges” in relation to specifically targeted cognitive biases (e.g., anchoring and social desirability effects) influenced user deliberation and responses. These studies highlight how strategic interface design can help to enhance reflection and reduce passive reception of information.

For example specific to AI system interfaces, design elements might vary according to stakeholder type. An interface designed to reduce physicians’ overreliance on an AI model estimating a patient’s 1-year survival post-intervention might include brief questions or a checklist encouraging physicians to document which other clinical, psychosocial, or environmental factors or additional expert opinions they have consulted in order to corroborate (or challenge) the AI’s estimate. Complementarily, a patient-facing interface for the same tool may contextualize the numerical survival estimate within a more holistic values clarification exercise asking patients to circle one or more treatment goals influencing their decisions, encouraging reflective, value-based decision making. Building in such reflexivity metrics could not only help to nudge users away from overreliance on AI tools but also evaluate impacts on clinical decision making in practice, both within and beyond clinical trial contexts.

However, interfaces are not the only tools available with this capacity. Conceptualizing how an AI system might fit into clinical flow in ways that encourage deliberation among clinical teams may also help to reduce potential for overreliance^91^. Situational and logistical factors could be considered, such as setting (e.g., the collective use of an AI tool during medical review board vs. individually in a physician’s office), timing (before or after treatment candidacy), and information access (direct-to-patient versus physician-privileged communication of results). Integration of AI with other existing clinical technologies may also alter outcomes of using AI tools by broadening information that is integrated into decision making^92^. Organizational aspects may include training, supervision, handover, and information flow across members of the clinical team^91^.

These insights discussed above represent only the tip of the iceberg of factors that may potentially be coordinated to positively influence decision quality and outcomes using AI. They have been identified and often widely discussed in fields as diverse as decision science, behavioral economics, human factors, psychology, political science, robotics, and others. However, few of these insights have yet been integrated into AI systems design or systematically tested in clinical trials to proactively shape how AI is used.

## Conclusion

We echo others’ calls that before AI tools are “released into the wild,” we must better understand their outcomes and impacts in the hands of imperfect human actors by testing at least some of them according to a risk-based approach in clinical trials that reflect their intended use settings. We advance this proposal by drawing attention to the need to empirically identify and test how specific user biases and decision contexts shape how AI tools are used in practice and influence patient outcomes. We propose that VSD can be used to strategize human-machine interfaces in ways that encourage critical reflection, mitigate bias, and reduce overreliance on AI systems in clinical decision making. We believe this approach can help to reduce some of the burdens on physicians to figure out on their own (with only basic training or knowledge about AI) the optimal role of AI tools in decision making by embedding a degree of bias mitigation directly into AI systems and interfaces.

## References

[1] Obermeyer Z, Emanuel EJ (2016). Predicting the future—big data, machine learning, and clinical medicine. N. Engl. J. Med..

[2] Klugman CM, Gerke S (2022). Rise of the bioethics AI: curse or blessing?. Am. J. Bioeth..

[3] U.S. Food and Drug Administration. Artificial intelligence/machine learning (AI/ML)-based software as a medical device (SaMD) action plan. (2021).

[4] Commission E. Laying Down Harmonised Rules on Artificial Intelligence (Artificial Intelligence Act) and Amending Certain Union Legislative Acts. European Commission (Brussels, 21.4.2021).

[5] Jobin A, Ienca M, Vayena E (2019). The global landscape of AI ethics guidelines. Nat. Mach. Intell..

[6] Chen T, Guestrin C. Proceedings of the 22nd ACM SIGKDD international conference on knowledge discovery and data mining. 2016. 10.1145/2939672.2939765 PMC5508051 28713636

[7] Markus AF, Kors JA, Rijnbeek PR (2021). The role of explainability in creating trustworthy artificial intelligence for health care: a comprehensive survey of the terminology, design choices, and evaluation strategies. J. Biomed. Inform..

[8] Babic B, Gerke S, Evgeniou T, Cohen IG (2021). Beware explanations from AI in health care. Science.

[9] U.S. Food and Drug Administration. Clinical Decision Support Software–Guidance for Industry and Food and Drug Administration Staff. (2022).

[10] U.S. Food and Drug Administration. U.S. Federal Food, Drug, and Cosmetic Act. (2018).

[11] Gerke S (2021). Health AI for good rather than evil? the need for a new regulatory framework for AI-based medical devices. Yale J. Health Policy, Law, Ethics.

[12] Gerke S, Babic B, Evgeniou T, Cohen IG (2020). The need for a system view to regulate artificial intelligence/machine learning-based software as medical device. NPJ Digit. Med..

[13] Nielsen J, Molich R (1990). Heuristic evaluation of user interfaces. Proc. SIGCHI Conf. Hum. factors Comput. Syst..

[14] Wu E (2021). How medical AI devices are evaluated: limitations and recommendations from an analysis of FDA approvals. Nat. Med..

[15] Price W.N. II. Medical AI and contextual bias. *Harvard Journal of Law and Technology* **33**, 2019.

[16] Babic B, Gerke S, Evgeniou T, Cohen IG (2019). Algorithms on regulatory lockdown in medicine. Science.

[17] Ansell DA, McDonald EK (2015). Bias, black lives, and academic medicine. N. Engl. J. Med..

[18] Kostick-Quenet KM (2022). Mitigating racial bias in machine learning. J. Law Med. Ethics.

[19] Blumenthal-Barby, J. S. Good ethics and bad choices: the relevance of behavioral economics for medical ethics. (MIT Press, 2021).

[20] Kahneman D., Slovic S. P., Slovic P. & Tversky A. Judgment under uncertainty: heuristics and biases. (Cambridge university press, 1982). 10.1126/science.185.4157.1124 17835457

[21] Pillutla MM, Malhotra D, Murnighan JK (2003). Attributions of trust and the calculus of reciprocity. J. Exp. Soc. Psychol..

[22] Corriveau KH (2009). Young children’s trust in their mother’s claims: longitudinal links with attachment security in infancy. Child Dev..

[23] Fett A-K (2016). Learning to trust: trust and attachment in early psychosis. Psychol. Med..

[24] Butler JK, Cantrell RS (1984). A behavioral decision theory approach to modeling dyadic trust in superiors and subordinates. Psychol. Rep..

[25] Mayer RC, Davis JH, Schoorman FD (1995). An integrative model of organizational trust. Acad. Manag. Rev..

[26] Grover SL, Hasel MC, Manville C, Serrano-Archimi C (2014). Follower reactions to leader trust violations: A grounded theory of violation types, likelihood of recovery, and recovery process. Eur. Manag. J..

[27] Banaji M. R. & Gelman S. A. Navigating the social world: what infants, children, and other species can teach us. (Oxford University Press; 2013).

[28] Fawcett C (2022). Kids attend to saliva sharing to infer social relationships. Science.

[29] Kaufmann L, Clément F (2014). Wired for society: cognizing pathways to society and culture. Topoi.

[30] Vickery J (2022). Challenges to evidence-informed decision-making in the context of pandemics: qualitative study of COVID-19 policy advisor perspectives. BMJ Glob. Health.

[31] Muñoz KA (2021). Pressing ethical issues in considering pediatric deep brain stimulation for obsessive-compulsive disorder. Brain Stimul..

[32] Hampson G, Towse A, Pearson SD, Dreitlein WB, Henshall C (2018). Gene therapy: evidence, value and affordability in the US health care system. J. Comp. Eff. Res..

[33] Wang, Z. J. & Busemeyer, J. R. Cognitive choice modeling. (MIT Press, 2021).

[34] Menon T, Blount S (2003). The messenger bias: a relational model of knowledge valuation. Res. Organ. Behav..

[35] Howard, J. Bandwagon effect and authority bias. Cognitive Errors and Diagnostic Mistakes. 21–56 (Springer; 2019).

[36] Slovic P (1995). The construction of preference. Am. Psychol..

[37] Levine LJ, Lench HC, Karnaze MM, Carlson SJ (2018). Bias in predicted and remembered emotion. Curr. Opin. Behav. Sci..

[38] Christman, J. The politics of persons: Individual autonomy and socio-historical selves. (Cambridge University Press, 2009).

[39] Samuelson W, Zeckhauser R (1988). Status quo bias in decision making. J. Risk Uncertain..

[40] Hardisty DJ, Appelt KC, Weber EU (2013). Good or bad, we want it now: fixed‐cost present bias for gains and losses explains magnitude asymmetries in intertemporal choice. J. Behav. Decis. Mak..

[41] Alon-Barkat, S. & Busuioc, M. Decision-makers processing of ai algorithmic advice: automation bias versus selective adherence. https://arxiv.org/ftp/arxiv/papers/2103/2103.02381.pdf (2021).

[42] Bond RR (2018). Automation bias in medicine: The influence of automated diagnoses on interpreter accuracy and uncertainty when reading electrocardiograms. J. Electrocardiol..

[43] Cummings, M. L. Automation bias in intelligent time critical decision support systems. *Decision Making in Aviation.* 289–294 (Routledge, 2017).

[44] Jussupow E, Spohrer K, Heinzl A, Gawlitza J (2021). Augmenting medical diagnosis decisions? An investigation into physicians’ decision-making process with artificial intelligence. Inf. Syst. Res..

[45] Skitka LJ, Mosier KL, Burdick M (1999). Does automation bias decision-making?. Int. J. Hum. Comput. Stud..

[46] Dijkstra JJ, Liebrand WB, Timminga E (1998). Persuasiveness of expert systems. Behav. Inf. Technol..

[47] Logg JM, Minson JA, Moore DA (2019). Algorithm appreciation: people prefer algorithmic to human judgment. Organ. Behav. Hum. Decis. Process..

[48] Furnham A, Boo HC (2011). A literature review of the anchoring effect. J. Socio-Econ..

[49] Diab DL, Pui SY, Yankelevich M, Highhouse S (2011). Lay perceptions of selection decision aids in US and non‐US samples. Int. J. Sel. Assess..

[50] Dietvorst BJ, Simmons JP, Massey C (2015). Algorithm aversion: people erroneously avoid algorithms after seeing them err. J. Exp. Psychol. Gen..

[51] Promberger M, Baron J (2006). Do patients trust computers?. J. Behav. Decis. Mak..

[52] Gaube S (2021). Do as AI say: susceptibility in deployment of clinical decision-aids. NPJ Digit. Med..

[53] Mosier, K. L, Skitka, L.J., Burdick, M. D. & Heers, S.T. Automation bias, accountability, and verification behaviors. Proceedings of the Human Factors and Ergonomics Society Annual Meeting. pp. 204–208 (SAGE Publications Sage CA, Los Angeles, CA, 1996).

[54] Wickens CD, Clegg BA, Vieane AZ, Sebok AL (2015). Complacency and automation bias in the use of imperfect automation. Hum. Factors.

[55] Li D, Kulasegaram K, Hodges BD (2019). Why we needn’t fear the machines: opportunities for medicine in a machine learning world. Acad. Med..

[56] Paranjape K, Schinkel M, Panday RN, Car J, Nanayakkara P (2019). Introducing artificial intelligence training in medical education. JMIR Med. Educ..

[57] Park SH, Do K-H, Kim S, Park JH, Lim Y-S (2019). What should medical students know about artificial intelligence in medicine?. J. Educ. Eval. Health Prof..

[58] Leavy, S., O’Sullivan, B. & Siapera, E. Data, power and bias in artificial intelligence. https://arxiv.org/abs/2008.07341 (2020).

[59] Goyal M, Knackstedt T, Yan S, Hassanpour S (2020). Artificial intelligence-based image classification methods for diagnosis of skin cancer: challenges and opportunities. Comput. Biol. Med..

[60] Loftus TJ (2020). Artificial intelligence and surgical decision-making. JAMA Surg..

[61] Rudin C (2019). Stop explaining black box machine learning models for high stakes decisions and use interpretable models instead. Nat. Mach. Intell..

[62] Kelly CJ, Karthikesalingam A, Suleyman M, Corrado G, King D (2019). Key challenges for delivering clinical impact with artificial intelligence. BMC Med..

[63] Yu K-H, Beam AL, Kohane IS (2018). Artificial intelligence in healthcare. Nat. Biomed. Eng..

[64] Cowen MBCrRW. Is Human-On-the-Loop the Best Answer for Rapid Relevant Responses? 2021. https://www.japcc.org/essays/is-human-on-the-loop-the-best-answer-for-rapid-relevant-responses/ (accessed August 23 2022).

[65] Man, Y., Lundh, M. & Porathe, T. Seeking harmony in shore-based unmanned ship handling: from the perspective of human factors, what is the difference we need to focus on from being onboard to onshore? Human Factors in Transportation. 81–90 (CRC Press, 2016).

[66] Katznelson G, Gerke S (2021). The need for health AI ethics in medical school education. Adv. Health Sci. Educ..

[67] Grunhut J, Marques O, Wyatt AT (2022). Needs, challenges, and applications of artificial intelligence in medical education curriculum. JMIR Med. Educ..

[68] Doherty TS, Carroll AE (2020). Believing in overcoming cognitive biases. AMA J. Ethics.

[69] Friedman, B. & Nissenbaum, H. Bias in computer systems. Computer Ethics. 215–232 (Routledge, 2017).

[70] Introna LD, Nissenbaum H (2000). Shaping the web: why the politics of search engines matters. Inf. Soc..

[71] Friedman B., Kahn P. H., Borning A. & Huldtgren A. Value sensitive design and information systems. Early engagement and new technologies: opening up the laboratory. 55–95 (Springer, 2013).

[72] Flanagan M, Howe DC, Nissenbaum H (2005). Values at play: design tradeoffs in socially-oriented game design. Proc. SIGCHI Conf. Hum. factors Comput. Syst..

[73] Sengers P, Boehner K, David S, Kaye JJ (2005). Reflective design. Proc. 4th Decenn. Conf. Crit. Comput.: sense sensibility.

[74] DiSalvo C. Adversarial design: Mit Press; 2015.

[75] Agre, P. & Agre, P. E. Computation and human experience. (Cambridge University Press,1997).

[76] Friedman B, Kahn PH (1992). Human agency and responsible computing: implications for computer system design. J. Syst. Softw..

[77] Nissenbaum H (1996). Accountability in a computerized society. Sci. Eng. Ethics.

[78] Floridi L, Cowls J, King TC, Taddeo M (2020). How to design AI for social good: seven essential factors. Sci. Eng. Ethics.

[79] Dolan P (2012). Influencing behaviour: the mindspace way. J. economic Psychol..

[80] Kosters M, Van der Heijden J (2015). From mechanism to virtue: evaluating nudge theory. Evaluation.

[81] Smith, H. S. et al. A review of the MINDSPACE framework for nudging health promotion during early stages of the COVID-19 Pandemic. *Population Health Management,* 2022. 10.1089/pop.2021.0269 35353613

[82] Blumenthal-Barby JS (2012). Between reason and coercion: ethically permissible influence in health care and health policy contexts. Kennedy Inst. Ethics J..

[83] Hausman DM, Welch B (2010). Debate: to nudge or not to nudge. J. Polit. Philos..

[84] Sunstein C. R. Why nudge?: The politics of libertarian paternalism: Yale University Press; 2014.

[85] Witteman HO (2021). Systematic development of patient decision aids: an update from the IPDAS collaboration. Med. Decis. Mak..

[86] Dressel J, Farid H (2018). The accuracy, fairness, and limits of predicting recidivism. Sci. Adv..

[87] Zhang W, Yang T, Tangi Perrault S (2021). Nudge for reflection: more than just a channel to political knowledge. Proc. 2021 CHI Conf. Hum. Factors Comput. Syst..

[88] Weinmann C (2018). Measuring political thinking: development and validation of a scale for “deliberation within”. Polit. Psychol..

[89] Harbach M, Hettig M, Weber S, Smith M (2014). Using personal examples to improve risk communication for security & privacy decisions. Proc. SIGCHI Conf. Hum. factors Comput. Syst..

[90] Menon S, Zhang W, Perrault ST (2020). Nudge for deliberativeness: how interface features influence online discourse. Proc. 2020 CHI Conf. Hum. Factors Comput. Syst..

[91] Sujan M., Furniss D., Hawkins R. D. & Habli, I. Human factors of using artificial intelligence in healthcare: challenges that stretch across industries. Safety-Critical Systems Symposium: York; 2020.

[92] Sujan, M. et al. Human factors challenges for the safe use of artificial intelligence in patient care. *BMJ Health Care Inform.* **26**, e100081 (2019). 10.1136/bmjhci-2019-100081 PMC7252977 31780459

